# Broad Ligament Hernia Presenting as Acute Appendicitis: A Case Report

**DOI:** 10.7759/cureus.92500

**Published:** 2025-09-16

**Authors:** Amog Prakash, Fatima Alhammadi, Sara Salim, Fatima M Alhashimi, Maliha Jaffar, Samyuktha Harikrishnan, Faisel Ikram, Roger Gerjy, Sara AlBastaki

**Affiliations:** 1 Internal Medicine, College of Medicine, Mohammed Bin Rashid University of Medicine and Health Sciences, Dubai, ARE; 2 Medicine, College of Medicine, Mohammed Bin Rashid University of Medicine and Health Sciences, Dubai, ARE; 3 General Surgery, Mediclinic City Hospital, Dubai, ARE; 4 Medicine, Gulf Medical University, Ajman, ARE

**Keywords:** appendicitis, broad ligament hernia, diagnostic laparoscopy, herniated intestine, intestinal, luminal obliteration, lymphoid hyperplasia, obstruction

## Abstract

Internal hernias, although rare, can present as surgical emergencies in certain instances, depending on the patient's symptomatology and clinical features. Broad ligament hernia is considered a rare type of internal hernia. Acute appendicitis is a condition that is caused by the inflammation of the vermiform appendix in response to a fecalith or lymphoid aggregation. Although stemming from differing etiologies and pathophysiological mechanisms, the conditions mentioned above can present similar signs and symptoms in clinical practice. In this case, we present a 37-year-old previously healthy woman who presented with chief complaints of abdominal pain, with features consistent with acute appendicitis, wherein a CT of the abdomen showed an appendix of 7.5 mm with no other findings. However, she was incidentally found to have a broad ligament hernia intraoperatively during a diagnostic laparoscopy. This report describes the patient's management laparoscopically. The presentations of acute appendicitis and broad ligament hernia can be similar and pose a diagnostic challenge to many physicians, as described in this case. Thus, it is significant to highlight the differences to avoid possible complications. Due to the presence of a diagnostic dilemma, we believe that a laparoscopy would be the best option for such patients for diagnostic and therapeutic purposes.

## Introduction

Broad ligament hernia is a relatively rare type of hernia that occurs when segments of the small intestine protrude through certain fenestrations in the broad ligament [1]. These defects can either be unilateral or bilateral and congenital or secondary, wherein the former and latter result as a consequence of peritoneal defects and causes such as trauma, surgery, pregnancy, and spontaneous rupture of pelvic cystic lesions, respectively [2]. There are primarily three types of broad ligament hernia defects: type 1 is a defect through the entire broad ligament, type 2 is a defect through the mesovarium and mesosalpinx above the round ligament, and type 3 is a defect through the mesoligamentum teres, between the broad and round ligaments [3].

On the other hand, acute appendicitis is an inflammation of the vermiform appendix, one of the most common surgical conditions, predominantly in children and young adults, which often presents with features of abdominal pain, anorexia, nausea, and vomiting [4]. Patients with an internal hernia can present with similar symptoms to those of acute appendicitis, depending on where the hernia is located, which in turn can pose a deal of confusion to differentiate between the two [5]. In this case report, we present a 37-year-old Australian woman who presented to a tertiary care hospital with features consistent with acute appendicitis but who was incidentally found to have a broad ligament hernia through a defect found during surgery.

## Case presentation

A 37-year-old Australian female was transferred via air ambulance from a hospital in Baghdad, Iraq, to our institution, a multicenter tertiary care hospital in Dubai, United Arab Emirates (UAE), with a clinical suspicion of acute appendicitis. Earlier that morning, the patient reported having generalized cramping abdominal pain along with anorexia, nausea, and multiple episodes of non-bilious, non-bloody vomiting. However, the patient denied any fever, diarrhea, or constipation. She had no underlying co-morbidities that warranted regular medical therapy. However, she had a past surgical history of a dacryocystorhinostomy three years ago and liposuction on her legs five years ago. She had never smoked or used illicit drugs, and drank alcohol socially. No relevant family history was reported.

On examination, the patient was hemodynamically stable. The abdomen was soft and non-distended, with tenderness in the right lower quadrant. No rebound tenderness, guarding, or any other special signs, such as Rovsing, psoas, or obturator signs, were exhibited clinically. Baseline laboratory investigations were taken on the patient's arrival. C-reactive protein, complete blood count, and renal function tests were unremarkable (Table 1). A contrast-enhanced CT scan of the abdomen and pelvis revealed an appendix 7.5 mm in diameter, with no other remarkable findings. 

**Table 1:** Preoperative laboratory values for the patient

Parameters	Patient Value	Reference range
Hemoglobin	11.7	11.6-15 g/dL
White blood cell count	7,500	4,500-11,000 cells/microL
Creatinine	58	53 to 97.2 µmol/L
C-reactive protein	2.7	<1.0 mg/dL

Due to her presenting symptoms, equivocal physical exam, and imaging findings, she was admitted to the surgical ward in order to undergo a diagnostic laparoscopy under general anesthesia to achieve the goal of symptom relief and prevention of further and recurrent complications.

**Figure 1:**
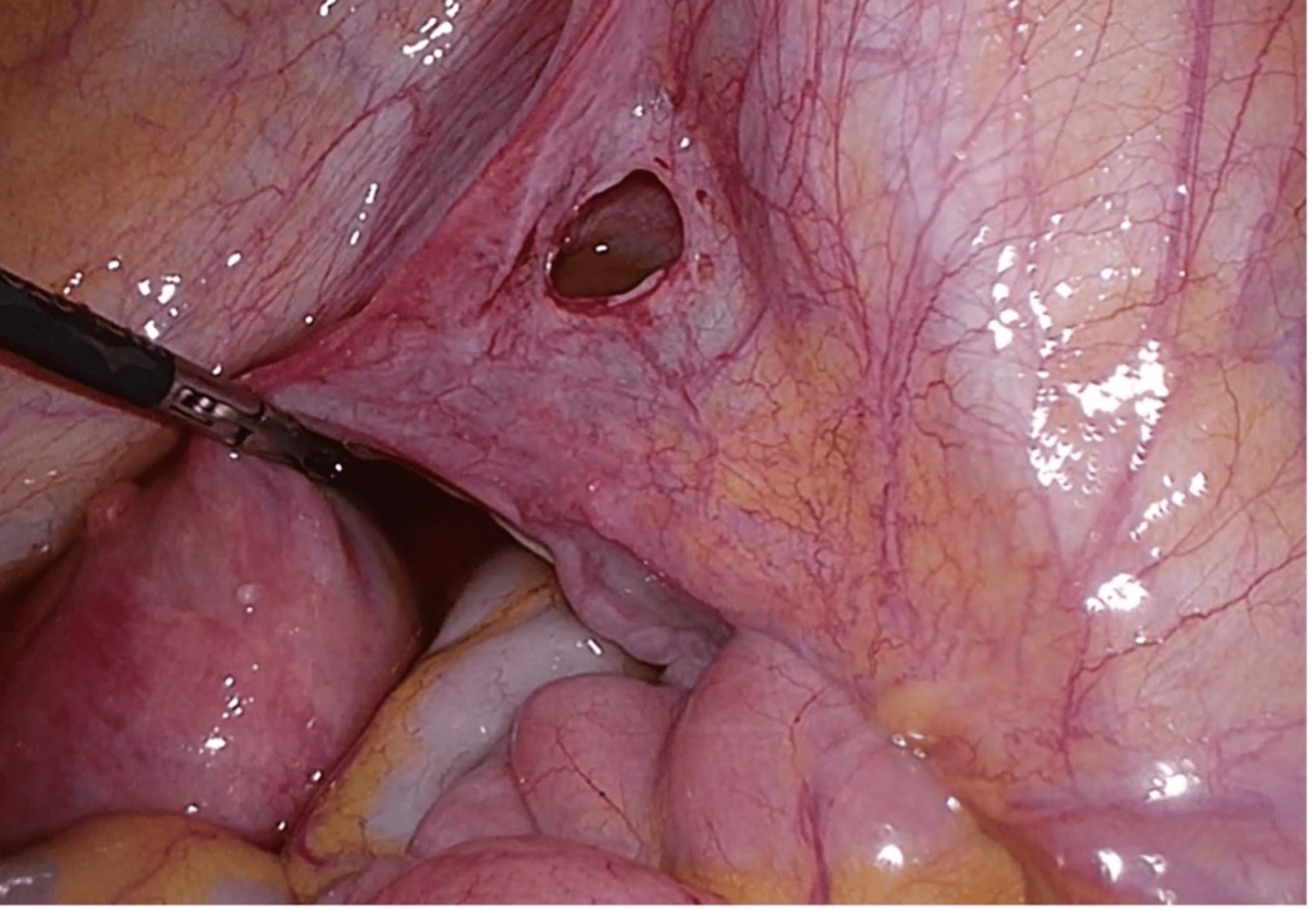
Incidental intraoperative finding of a broad ligament hernia situated above the round ligament, 2.5 cm in size, with evidence of recent herniation with hyperemic edges.

Interestingly, during the procedure, an incidental finding of a right-sided, type II broad ligament hernia was detected, being 2.5 cm in size, with evidence of recent herniation and reduction of small bowel with hyperemia at the hernial edges (Figure 1). The small bowel was grossly normal, with a hyperemic patch in the terminal ileum, suggesting a recent herniation in the broad ligament. There was a mildly hyperemic appendix in the right iliac fossa, which was subsequently removed in order to prevent future bouts of abdominal pain. Microscopic sections of the appendix showed dilation of the lumen marked by tenacious mucinous secretion with marked lymphoid hyperplasia and partial luminal obliteration. A broad ligament defect closure with continuous suture followed this.

The patient was discharged two days post surgery, wherein she showed great signs of recovery and did not have any significant postoperative complications.

## Discussion

Broad ligament hernia is also known as the Allen and Masters syndrome since they were the first to publish a case series on this in 1955 [6]. Hernial orifices can be congenital or acquired. Congenital broad ligament defects are generally bilateral, whereas acquired defects are primarily unilateral. Causes of acquired defects are previous surgery, pregnancy, birth-related trauma, or previous pelvic inflammatory disease, which increases the intra-abdominal pressure [7]. Due to the growing popularity of surgical procedures like gastric bypass, the overall incidence of internal hernias has recently been increasing [8]. Broad ligament hernia accounts for 4% of all internal hernias [9]. Due to broad ligament hernia being a rare female-specific phenomenon with a presentation similar to that of acute appendicitis and other causes of acute abdomen, as in our case, it can be considered a diagnostic challenge to the treating team [1].

In the context of clinical diagnostic challenges differentiating between acute appendicitis and internal hernia, the insightful work of Fousekis et al. sheds light on a notable case of De Garengeot's hernia accompanied by acute appendicitis [10]. They emphasize the importance of considering acute appendicitis within a hernia sac as a reasonable differential diagnosis, particularly in patients presenting with clinical manifestations suggestive of an incarcerated hernia in the femoral region. In a similar clinical scenario, Fuentes-Diaz et al. reported the case of a 19-year-old male patient who presented with features suggestive of acute appendicitis; however, subsequent appendectomy revealed unanticipated ischemic and necrotic changes in a bowel segment, prompting the necessity for a laparotomy [11]. During the exploratory procedure, the surgical team uncovered a distinctive internal hernia characterized by portions of ischemic and necrotic jejunum, coupled with another bowel segment exhibiting firm adherence to the mesentery root. This unique internal hernia was identified as a giant Meckel diverticulum.

In terms of diagnostic methods, a broad ligament hernia is not a condition that is easily seen radiologically. A CT scan can diagnose a broad ligament hernia only when the mesenteric vessels of the herniated intestine are seen penetrating through the broad ligament defect [12]. Multimodal imaging techniques such as CT scans, ultrasound, and plain radiographs are most used when obstruction or strangulation is suspected [13]. As highlighted in previous studies, a CT scan and an ultrasound were successful 36% and 20% of the time in diagnosing a broad ligament hernia, respectively [3]. Even in our case, the CT scan failed to highlight the presence of a broad ligament hernia, which was found incidentally during the diagnostic laparoscopy. The resemblance in clinical presentation and the intrinsic complexities in radiological diagnosis contributed to the dilemma of distinguishing between these two conditions.

Based on Cilley’s classification, our case involves a type 2 broad ligament hernia, where the defect is situated above the round ligament, between the mesovarium and the mesosalpinx [14]. 

Because of the rarity of broad ligament hernia, a standard procedure for repair has yet to be determined. Given the advances in surgical management in terms of minimal access procedures, laparoscopic surgery has become an essential tool when it comes to cases like ours, to avoid unwarranted complications. We feel a laparoscopic approach would be best to tackle a diagnostic dilemma, as in our case, as it could be both diagnostic and therapeutic. Robust studies are needed to prove the differences/similarities in both conditions and to find imaging or other investigation modalities that may be able to differentiate between the two in cases of ambiguity.

## Conclusions

This case represents a rare instance where acute appendicitis was accompanied by the incidental discovery of a broad ligament hernia intraoperatively. Due to the similar presentation and clinical features found on physical exam, there can be a degree of confusion and difficulty in differentiating between an acute appendicitis and an uncomplicated internal hernia in some cases. Therefore, an internal hernia should be included in the differential diagnosis of a suspected acute appendicitis and vice versa. Secondly, in case of a diagnostic dilemma between the two, wherein history, physical exam, laboratory, and imaging results remain inconclusive, a laparoscopic approach can be employed for diagnostic and therapeutic purposes. Large-scale clinical studies are therefore warranted.
